# Pore shape-reflecting morphosynthesis of lithium niobium oxide *via* mixed chloride flux growth in the presence of mesoporous silica†

**DOI:** 10.1039/c9na00097f

**Published:** 2019-04-10

**Authors:** Minoru Sohmiya, Shinya Umehara, Shinpei Enomoto, Yusuke Ide, Tomohiko Okada, Yoshiyuki Sugahara, Makoto Ogawa

**Affiliations:** Department of Materials and Life Science, Faculty of Science and Technology, Seikei University 3-3-1 Kichijojikitamachi Musashino-shi Tokyo 180-8633 Japan minoru.sohmiya@st.seikei.ac.jp minoru.sohmiya@gmail.com; Department of Earth Sciences, Waseda University 1-6-1 Nishiwaseda, Shinjuku-ku Tokyo 169-8050 Japan; Kagami Memorial Laboratory for Materials Science and Technology (Zaiken), Waseda University 2-8-26 Nishiwaseda, Shinjuku-ku Tokyo 169-0051 Japan; Graduate School of Creative Science and Engineering, Waseda University 1-6-1 Nishiwaseda, Shinjuku-ku Tokyo 169-8050 Japan; International Center for Materials Nanoarchitectonics (MANA), National Institute for Materials Science (NIMS) 1-1 Namiki Tsukuba Ibaraki 305-0044 Japan; Department of Chemistry and Materials Engineering, Faculty of Engineering, Shinshu University 4-17-1 Wakasato Nagano 380-8553 Japan; Department of Applied Chemistry, School of Advanced Science and Engineering, Waseda University 3-4-1 Ohkubo, Shinjuku-ku Tokyo 169-8555 Japan; School of Energy Science and Engineering, Vidyasirimedhi Institute of Science and Technology 555 Moo 1, Payupnai, Wangchan Rayong 21210 Thailand

## Abstract

A new synthesis method, “chloride flux growth in the rigid nanospace of mesoporous silica”, was developed to obtain lithium niobium oxide anisotropic nanoparticles. The morphologies reflect the pore size and shape of the used mesoporous silicas. This method has great potential for synthesizing size-tuned anisotropic nanoparticles of other complex metal oxides.

## Abstract 2

“Chloride flux growth in the rigid nanospace of mesoporous silica” was employed to produce lithium niobium oxide anisotropic nanoparticles.

## Graphical abstract

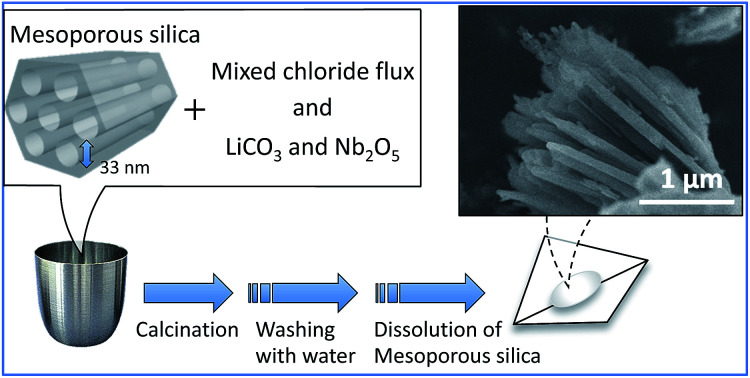

## Introduction

Synthesis of anisotropic nanoparticles remains a topic of interest from both the scientific and industrial viewpoints.^1,2^ Considerable efforts have been devoted to the development of synthesis methods for controlling their morphology (controlling the rate of facet growth),^1–6^ such as sol–gel processes, reverse micelle method, homogeneous precipitation, hydrothermal/solvothermal methods, and topochemical intermediate reactions. Another promising approach is the use of limited nanospaces as reaction vessels.^7–14^ The rigid, uniform, and size- and shape-tunable nanopores of mesoporous materials, including mesoporous silica, have often been utilized;^4,10,11^ precursors are adsorbed/deposited onto the pores from solution or through vapors, and sequentially converted into inorganic nanoparticles by post-treatment such as calcination. This method using a periodic porous material as a “template” provides nanoparticles with morphologies that reflect the pore size and shape; these may be nanoparticles,^8,15–18^ including nanowires,^15,19–24^ or inverse replicas, including mesoporous carbons.^25,26^

Flux growth is a well-known simple procedure for obtaining idiomorphic crystals with high crystallinity.^27^ Chloride flux growth, especially, is an inexpensive, environmentally friendly, non-hazardous procedure that requires moderately low temperature (LiCl–NaCl–KCl mixtures show a significantly low eutectic point of 346 °C (ref. 28)). Chloride flux is usually a poor solvent for metal oxides, however, and is thus difficult to use for anisotropic nanoparticle production.

**Scheme 1:**
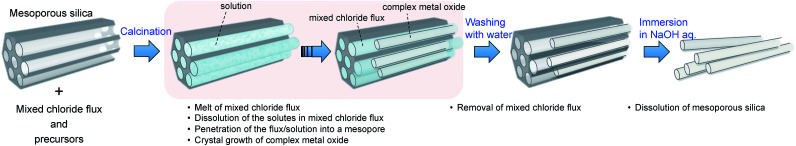
Schematic diagram of ideal “chloride flux growth in the rigid nanospace of mesoporous silica”.

Here we demonstrate a synthesis method combining the above two methods (Scheme 1) for the first time to our knowledge (although flux growth using mesoporous silica as a silica source was reported^29^). This new method “chloride flux growth in the rigid nanospace of mesoporous silica” was employed to produce lithium niobium oxide nanoparticles.

## Results and discussion

Mesoporous silicas, SBA-15, were synthesized based on the previous report.^30^ The pore size varied depending on the amount of 1,3,5-trimethylbenzene (TMB) added (Table 1) as shown by nitrogen adsorption/desorption isotherms (Fig. S1†). Mesoporous silicas are abbreviated as SBA-15(*x*), where *x* denotes the pore size (nm) estimated by the BJH method^31^ (Fig. S2†). A mixed chloride flux (NaCl, KCl, and LiCl, Na : K : Li = 9 : 36 : 55 in mol, eutectic point of 346 °C,^28^ which was checked by differential scanning calorimetry (DSC, data not shown)) and the solutes (Nb_2_O_5_ and Li_2_CO_3_, where Nb : Li = 1 : 3 in mol) were added to 0.50 g of mesoporous silica. The weight ratio of the solutes in the additives (the solutes and the mixed chloride flux) was 20%, and the volume of the additives was equal to the pore volume of the mesoporous silica (calculated from the density of each material; ESI†). Each mixture was beaten lightly and placed in a platinum crucible. The mixture was calcined at 550 °C in air for 10 h at a heating rate of 10 °C min^−1^, cooled down to 300 °C at a cooling rate of 10 °C min^−1^, and then cooled to room temperature with no control. The resulting crude product was washed repeatedly with deionized water until a negative AgNO_3_ test was obtained, and dried in air at 60 °C. The washed product was then immersed in a 1 M NaOH aqueous solution at 100 °C to dissolve the silica and dried in air at 60 °C.

**Table 1:** Characteristics of SBA-15 prepared in the present study

	Weight ratio of TMB to P123	BET surface area (m^2^ g^−1^)	Pore volume^a^ (mL g^−1^)	Pore size^b^ (nm)
SBA-15(9)	0	650	0.99	9.3
SBA-15(21)	0.5	720	2.0	21
SBA-15(33)	1	430	2.0	33

a Pore volumes are calculated from the adsorbed amount of N_2_ at a relative pressure of 0.99.

b Pore sizes are evaluated by the BJH method from the adsorption branch.

**Fig. 1:**
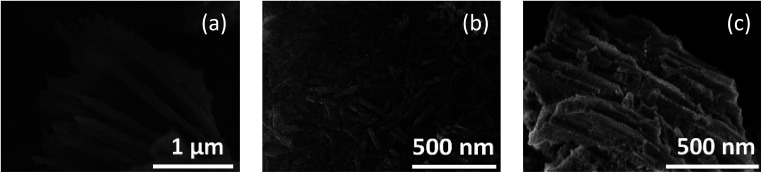
FE-SEM images of the products synthesized in the presence of SBA-15(33) (a), (21) (b), and (9) (c).

**Fig. 2:**
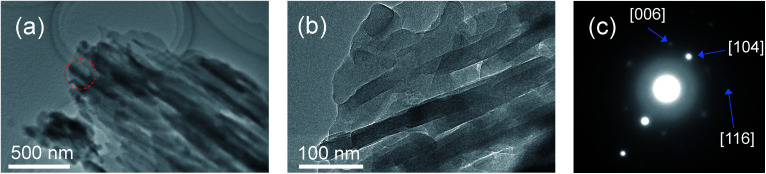
FE-TEM images (a) and (b) and the SA-ED pattern (c) of the product synthesized in the presence of SBA-15(33). The red circle in (a) indicates the selected area.

A field emission scanning electron micrograph (FE-SEM) and field emission transmission electron micrograph (FE-TEM) showed that the morphologies of the products reflected the pore shape and size of the mesopores when synthesized in the presence of SBA-15(33) and SBA-15(21) but not in the presence of SBA-15(9). Synthesis in the presence of SBA-15(33) produced bundles measuring 80–100 nm in diameter, which were composed of nanorods measuring 25–50 nm in diameter and 50 nm to 2 μm in length (Fig. 1a and 2), while synthesis in the presence of SBA-15(21) produced nanorods measuring *ca.* 25 nm in diameter and 200–400 nm in length (Fig. 1b and S3†). In the presence of SBA-15(9), on the other hand, agglomerates were observed (Fig. 1c).

**Fig. 3:**
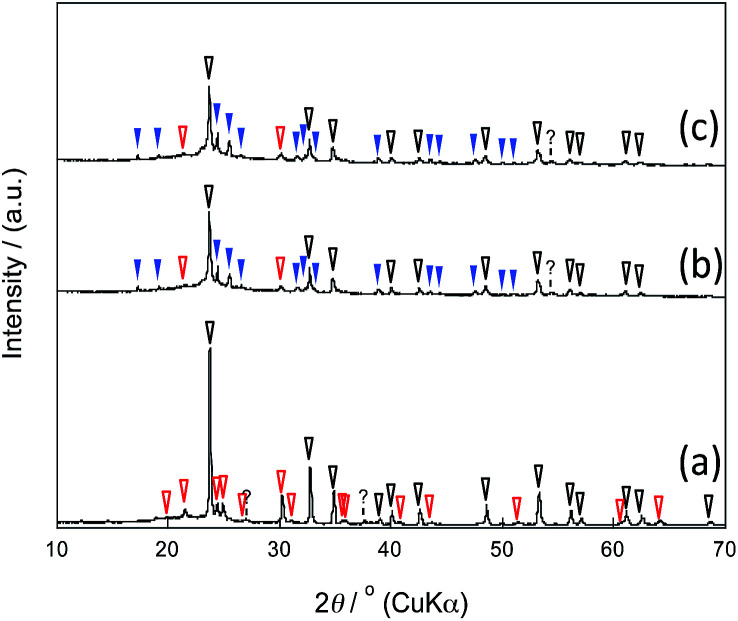
XRD patterns of the products synthesized in the presence of SBA-15(33) (a), (21) (b), and (9) (c); the marks white, red, and blue correspond to LiNbO_3_, LiNb_3_O_8_, and Nb_2_O_5_, respectively.

The X-ray diffraction (XRD) patterns showed that the mixed chloride flux growth in the presence of mesoporous silica provided rhombohedral LiNbO_3_ (space group *R*3*c*) as the main product (Fig. 3a–c); LiNb_3_O_8_ was obtained as a byproduct in the presence of SBA-15(33); LiNb_3_O_8_ and Nb_2_O_5_ were present as a byproduct and a remaining solute, respectively, in the presence of SBA-15(21) and SBA-15(9). The crystallite diameters estimated from the diffraction lines attributable to the (012), (104), and (110) planes of LiNbO_3_ (three strong diffraction lines) using the Scherrer equation were 40 ± 10 nm for the three diffraction lines of the three samples. This finding supports the evidence provided by the above FE-TEM images. Selected-area electron diffraction (SA-ED) patterns of the product synthesized in the presence of SBA-15(33) showed diffraction spots attributable to LiNbO_3_ (Fig. 2c), indicating that the bundles were composed of anisotropic nanoparticles of LiNbO_3_. We could not obtain the SA-ED patterns of the product synthesized in the presence of SBA-15(21), however; it collapsed immediately under electron beam irradiation, probably due to its smaller particle size and shape.

LiNbO_3_ nanoparticles, especially anisotropic nanoparticles including nanorods and nanowires, are of great interest due to their potential applications in such products as ferroelectric memory devices, piezoelectric devices, and optical sensors including bio-imaging probes such as second harmonic probes.^32–37^ LiNbO_3_ anisotropic nanoparticles were prepared by several methods: hydrothermal methods,^32,34^ solvothermal methods,^38^ solution-phase methods,^39^ and topochemical synthesis.^34,40^ Crystallization inside rigid, uniform pores of porous silicon^41^ and porous alumina^42^ was reported to provide the particles with pore shape-reflecting morphologies, but with dimensions of several hundred nm. Crystallization using mesoporous silica as a “template” has been reported;^43^ due to the material's thermal stability,^44^ only rapid, short-time heating of the precursors inside the mesopores at over 700 °C using an IR furnace can be conducted. Compared to this, the present method is simple and easy, and offers the potential to be employed for the synthesis of other complex oxides, which have been obtained *via* flux growth. The synthesis mechanism should nevertheless be clarified for purposes of improvement.

**Fig. 4:**
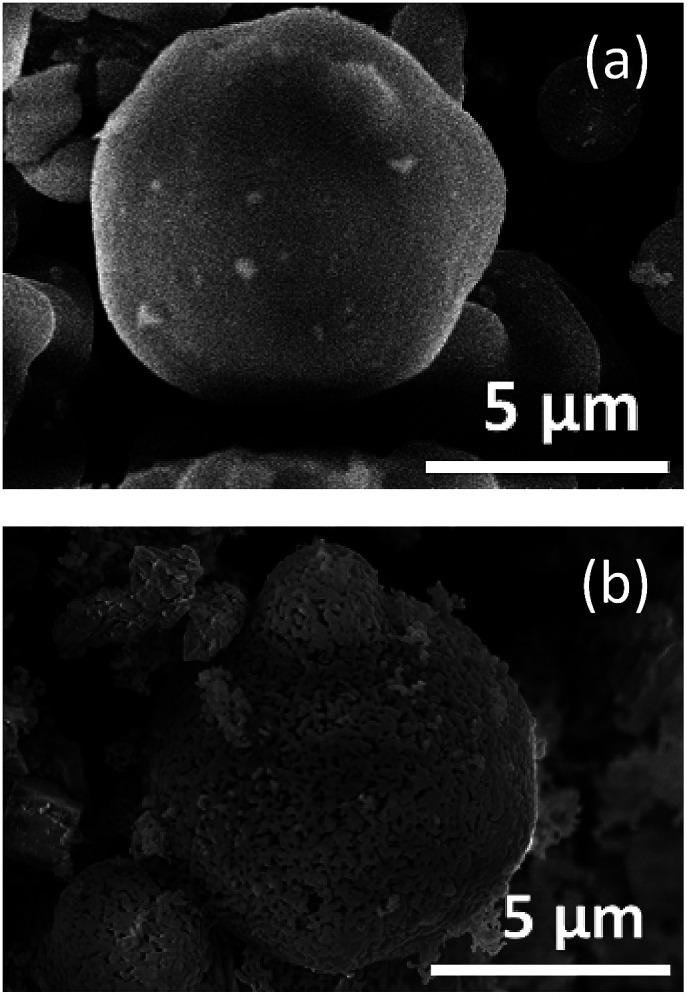
FE-SEM images of SBA-15 (a) and the sample calcined in the presence of SBA-15(33) after washing with water before immersion in hot NaOH aq. (b).

Fig. 4 shows the FE-SEM images of SBA-15(33) (Fig. 4a) and the sample calcined in the presence of SBA-15(33) after washing with water before immersion in hot NaOH aq. (Fig. 4b). After calcination and washing with water, the particle surface of SBA-15(33) appeared to have melted due to the reaction of SBA-15(33) with the flux. This is supported by the result showing that the calcination of the mixed chloride flux and SBA-15(33) (without the solutes) at 550 °C provided lithium silicates (Fig. S4†). The flux growth with only the mixed chloride flux and solutes (without mesoporous silica) at the same solute weight ratio (20%) provided Li_3_NbO_4_ and LiNbO_3_ (Fig. S5a†) as the main product and byproduct, respectively, but did not show the presence of solutes, indicating that the solutes, Nb_2_O_5_ and Li_2_CO_3_, were apparently dissolved in the mixed chloride flux at 550 °C. We also observed the dissolution behavior of the solutes in the mixed chloride flux at 500 °C using an optical microscope (Fig. S6†) to find that the mixed chloride flux melted before the solutes did.

Based on the above results, the synthesis mechanism is considered as follows. In the heating process, the mixed chloride flux melted at 346 °C and began to dissolve the precursor solutes and to react with the mesoporous silica to form lithium silicate. The resulting solution penetrated into the pores, probably driven by capillary condensation, which occurred at lower temperatures with smaller pores. Lithium niobium oxide then began to crystalize in the pores to form anisotropic nanoparticles with morphologies reflecting the pore size and shape. The reaction of mixed chloride flux with the template (to form lithium silicate) changed the composition of the solution, most notably the molar ratio of Li to Nb. This mechanism can explain the difficulty of obtaining lithium niobium oxides stoichiometrically and the presence of Nb_2_O_5_ when mesoporous silicas with smaller pore size, SBA-15(21) and (9), were used (Fig. 3b and c). Accompanying the formation of lithium silicate and crystallization of lithium niobium oxides, the Li ion concentration of the mixed chloride flux decreased to cause the eutectic point to rise. This could make it difficult for the solution to dissolve the remaining solutes and to penetrate into the pores, especially smaller ones. Consideration of a phase diagram of the Li_2_O–Nb_2_O_5_ system supports this hypothesis (ESI).†

To overcome such problems as collapsing templates or difficulties in obtaining stoichiometric products, the pore sizes and shapes of the mesoporous silica, the composition and mixing conditions of the flux, solutes, and mesoporous silica (templates), and the heating and cooling conditions should be optimized. The method described here might be improved, for example, by mixing the flux solution and mesoporous silica after heating them separately.

## Conclusions

Mixed chloride flux growth of lithium niobium oxide in the presence of mesoporous silica was achieved for the first time. The following reactions occurred to form pore shape-reflecting particles of lithium niobium oxide at above the eutectic point of the mixed chloride flux (346 °C): dissolution of the precursor solutes in the melt-mixed chloride flux; penetration of the mixed chloride flux/solution into the pores; crystal growth of lithium niobium oxide in the pores; and reaction of the lithium ion source with the mesoporous silica to form lithium silicate and an accompanying rise in the eutectic point. Under the present synthesis conditions, the morphologies of the products, with LiNbO_3_ as the main product, reflected the pore sizes and shapes of the mesoporous silicas; depending on the pore size (33 nm or 21 nm), the bundles comprised nanorods measuring 25–50 nm in diameter and 50 nm to 2 μm in length or nanorods measuring *ca.* 25 nm in diameter and 200–400 nm in length, respectively. Since chloride flux growth is applicable to the preparation of various complex oxides, this mixed chloride flux growth in the rigid nanospace of mesoporous silica is a promising procedure for obtaining size-tuned anisotropic nanoparticles of lithium niobium oxide and of other complex oxides.

## Conflicts of interest

There are no conflicts to declare.

## Supplementary Material

NA-001-C9NA00097F-s001
