# Supplementary material for: Pore shape-reflecting morphosynthesis of lithium niobium oxide via mixed chloride flux growth in the presence of mesoporous silica
Source: Nanoscale Adv. 2019 Apr 10;1(5):1726–30. doi: 10.1039/c9na00097f (PMC9419088; doi:10.1039/c9na00097f)
Supplement: NA-001-C9NA00097F-s001 [file NA-001-C9NA00097F-s001.pdf]

Electronic Supplementary Material (ESI) for Nanoscale Advances.
This journal is © The Royal Society of Chemistry 2019

Electronic Supporting Information (ESI)

Pore shape-reflecting morphosynthesis of lithium niobium oxide *via* mixed chloride flux growth in the presence of mesoporous silica

Minoru Sohmiya,*[a, b, c] Shinya Umehara,[d] Shinpei Enomoto,[c] Yusuke Ide,[d, e] Tomohiko Okada,[d, f] Yoshiyuki Sugahara[c, d, g] and Makoto Ogawa[b, d, h]

[a.] Department of Materials and Life Science, Faculty of Science and Technology, Seikei University, 3-3-1 Kichijojikitamachi, Musashino-shi, Tokyo 180-8633, Japan.

[b.] Department of Earth Sciences, Waseda University, 1-6-1 Nishiwaseda, Shinjuku-ku, Tokyo 169-8050, Japan.

[c.] Kagami Memorial Laboratory for Materials Science and Technology, Waseda University, 2-8-26 Nishiwaseda, Shinjuku-ku, Tokyo 169-0051, Japan.

[d.] Graduate School of Creative Science and Engineering, Waseda University, 1-6-1 Nishiwaseda, Shinjuku-ku, Tokyo 169-8050, Japan.

[e.] International Center for Materials Nanoarchitectonics (MANA), National Institute for Materials Science (NIMS), 1-1 Namiki, Tsukuba, Ibaraki 305-0044, Japan.

[f.] Department of Chemistry and Materials Engineering, Faculty of Engineering, Shinshu University, 4-17-1 Wakasato, Nagano 380-8553, Japan.

[g.] Department of Applied Chemistry, School of Advanced Science and Engineering, Waseda University, 3-4-1 Ohkubo, Shinjuku-ku, Tokyo 169-8555, Japan.

[h.] *School of Energy Science and Engineering, Vidyasirimedhi Institute of Science and Technology, 555 Moo 1, Payupnai, Wangchan, Rayong 21210, Thailnd.*

*Correspondence to: minoru.sohmiya@st.seikei.ac.jp or minoru.sohmiya@gmail.com

Table of Content

1. Experimental
2. Consideration on Phase diagram of the Li_2O-Nb_2O_5 system
3. Supporting Figures (Figs. S1 to S6)
4. References

1. Experimental

Materials

Tetraethoxysilane (TEOS) and 1,3,5-trimethylbenzene (TMB) were purchased from Tokyo Chemical Industry Co., Ltd. Poly(ethylene glycol)-block-poly(propylene glycol)-block-poly(ethylene glycol) (P123) was obtained from Sigma-Aldrich Co. LLC. LiCl was purchased from Wako Pure Chemicals Industries Co. NaCl, KCl, Nb_2O_5, Li_2CO_3, NaOH, and 35-37 wt% HCl, were obtained from Kanto Chemical Co., Inc. All the chemicals were used without further purification.

Sample preparation

Preparation of mesoporous silicas with various pore sizes (SBA-15)

Mesoporous silicas (SBA-15) with various pore sizes were synthesized by the method described in the previous report.[1] P123 (4.0 g), TMB (0, 2.0 or 4.0 g), HCl (20 mL), and deionized water (104 g) were mixed in a polypropylene vessel and stirred at 50°C to make a homogeneous solution, and 8.56 g of TEOS was then added slowly to the mixture under stirring. The mixture was stirred for 24 hours, transferred into a Teflon®-lined autoclave, and aged at 100°C for 72 hours. The white precipitates were then centrifuged and washed with deionized water and dried at 60°C for 1 day. Finally, the as-synthesized samples were calcined in air at 550°C for 6 hours. The products were abbreviated as SBA-15(*x*), where *x* denotes the pore size diameter calculated by the BJH method.

Preparation of lithium niobium oxide *via* flux growth in the presence of mesoporous silica

The following procedure is representative of synthesis in the presence of SBA-15(33). The volumes of the solutes and the mixed flux were adjusted (based on the density of each material) to the pore volume of mesoporous silica, SBA-15(*x*).

NaCl, KCl and LiCl were ground with a mortar and pestle, in a molar ratio of Na : K : Li = 9: 36 : 55. Subsequently, 209 mg of Nb_2O_5, 175 mg of Li_2CO_3, and 1540 mg of the mixed flux (weight ratio of the solutes in the additives = 20%) were ground with a mortar and pestle, 0.500 g of mesoporous silica, SBA-15(33), was added, and the mixture was then beaten lightly (Nb : Li = 1 : 3 in mol). After beating, the mixture was poured into a platinum crucible with a capacity of 30 cm^3 and calcined at 550°C in air for 10 h (heating rate = 10 ^{o}C min^{-1}), cooled down to 300°C at a cooling rate of 10 ^{o}C min^{-1}, and then cooled to room temperature. The resulting sample was washed with deionized water repeatedly until a negative $AgNO_3$ test was obtained, and dried in air at 60°C. The obtained powder was immersed in ca. 100 mL of 1 M NaOH aqueous solution at 100°C to dissolve the silicate and dried in air at 60°C.

Characterization

The nitrogen adsorption/desorption isotherms of the mesoporous silica samples were measured at 77 K with a BELSORP mini instrument (BEL Japan, Inc.). Prior to measurement, the samples were heated at 120 °C for 2 h under a nitrogen flow. The differential scanning calorimetry (DSC) curve for the mixed chloride flux was obtained with a Rigaku Thermo Plus DSC 8230L. The morphologies of the products were observed with a Hitachi S-5500 field emission scanning electron microscope (FE-SEM) and a JEOL JEM-2100 field emission scanning transmission electron microscope (FE-TEM). The selected-area electron diffraction (SA-ED) patterns were also obtained with a JEOL JEM-2100. XRD patterns of the solid products were recorded on a Rigaku SmartLab powder diffractometer equipped with monochromatic CuKα radiation operated at 30 mA and 40 kV. Optical microscopic images were obtained with a Nikon Eclipse E600 equipped with a temperature-controlled stage TMS94 (Linkam Scientific Instrument).

2. Consideration on Phase diagram of the Li_2O-Nb_2O_5 system

The phase diagram of the Li_2O-Nb_2O_5 system proposed by Svaasand *et al.*[2], which is a modified version of that presented by Reisman and Holtzberg[3], shows that both Li_3NbO_4 and $LiNbO_3$ are obtained in the Li_2CO_3 molar ratio range from 51% to below 75% and that both $LiNbO_3$ and $LiNb_3O_8$ are obtained in the range from above 25% to 47%. $LiNbO_3$ can be synthesized *via* mixed flux growth at 25% of the Li_2CO_3 molar ratio (corresponding to Nb : Li = 3:1 in mol; Fig. S4b), but single-phase Li_3NbO_4 was not obtained at 75% of the Li_2CO_3 molar ratio (corresponding to Nb : Li = 1 : 3 in mol; Fig. S4a), as mentioned above. The reaction conditions of mixed chloride flux growth conducted in this paper must reflect a different phase diagram, but they may exhibit similar tendencies.

3. Supporting Figures (Fig. S1-S6)

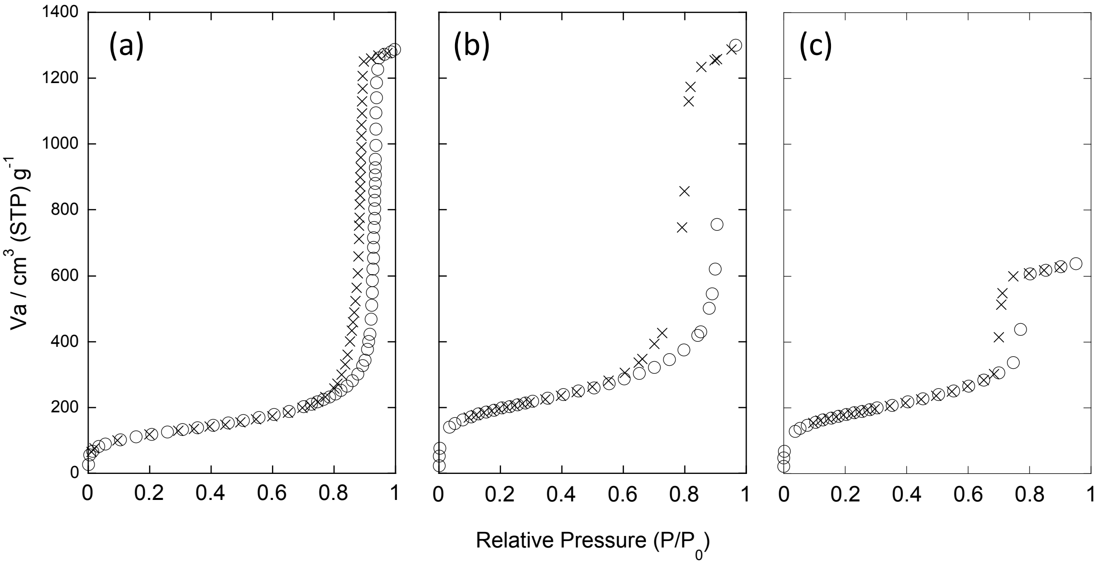

Fig. S1 Nitrogen adsorption/desorption isotherms of SBA-15 (33) (a), (21) (b), and (9) (c).

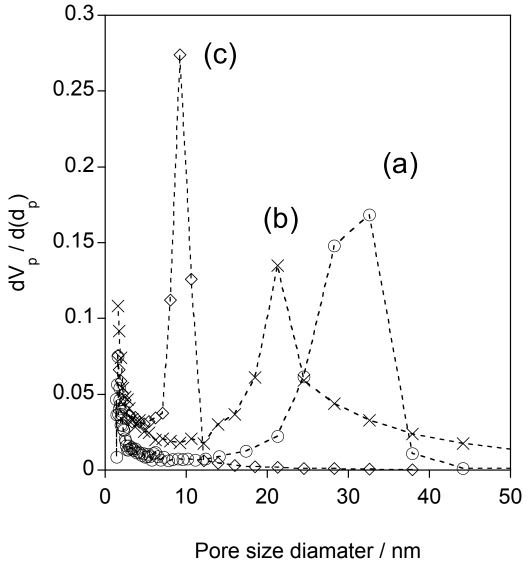

Fig. S2 Pore size distributions of SBA-15 (33) (a), (21) (b), and (9) (c), evaluated by the BJH method.

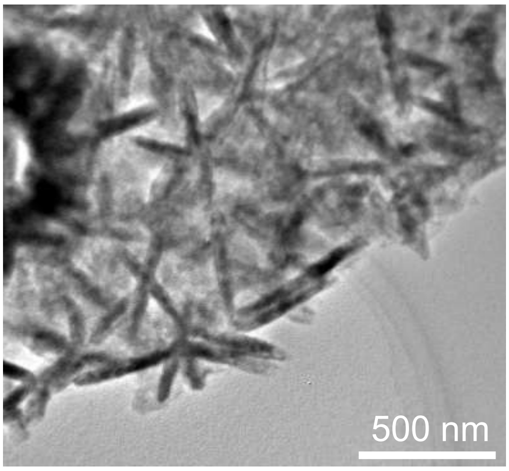

Fig. S3 FE-TEM image of the product synthesized in the presence of SBA-15(21).

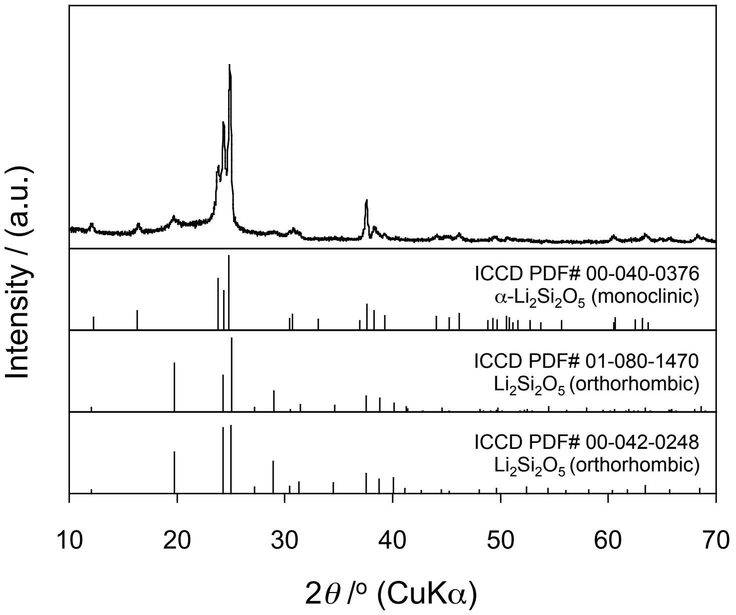

Fig. S4 XRD pattern of SBA-15 calcined with the mixed chloride flux.

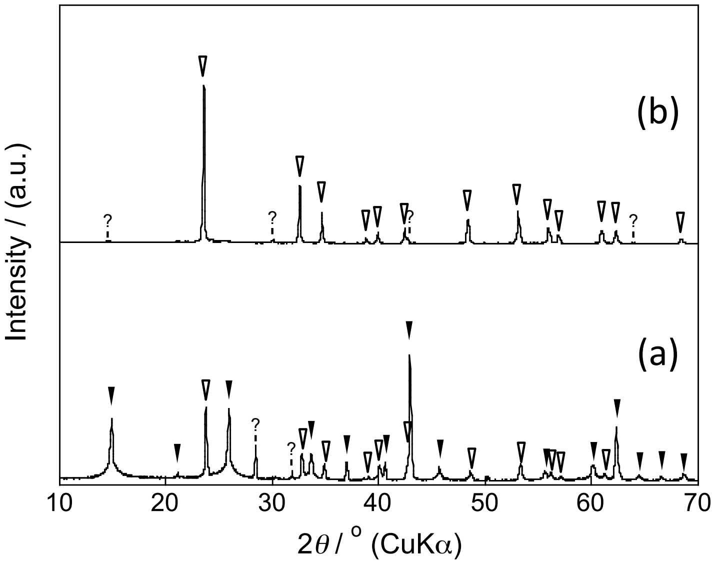

Fig. S5 XRD patterns of the products synthesized without SBA-15; Nb_2O_5:Li_2CO_3 = 1 : 3 (a) and = 3 : 1 (b). The marks white and black correspond to $LiNbO_3$ and Li_3NbO_4, respectively.

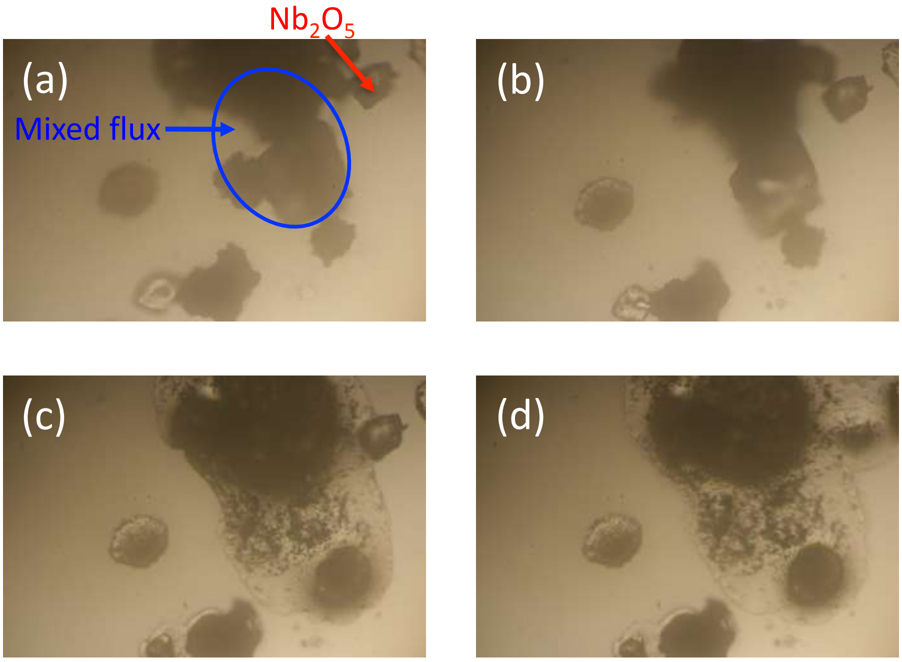

Fig. S6 Optical micrographs of the mixed chloride flux and the solutes: (a) at 20 °C, (b) at 500 °C for 0 min., (c) at 500 °C for 5 min., and (d) at 500 °C for 10 min.

4. References

1. D. Zhao, J. Feng, Q. Huo, N. Melosh, G. H. Fredrickson, B. F. Chmelka and G. D. Stucky, *Science*, 1998, **279**, 548-552.
2. L. O. Svaasand, M. Eriksrud, A. P. Grande and F. Mo, *J. Cryst. Growth*, 1973, **18**, 179-184.
3. A. Reisman and F. Holtzberg, *J. Am. Chem. Soc.*, 1958, **80**, 6503-6507.
